# PRDX1 negatively regulates bleomycin-induced pulmonary fibrosis via inhibiting the epithelial-mesenchymal transition and lung fibroblast proliferation in vitro and in vivo

**DOI:** 10.1186/s11658-023-00460-x

**Published:** 2023-06-02

**Authors:** Hu-Nan Sun, Chen-Xi Ren, Dong Hun Lee, Wei-Hao Wang, Xiao-Yu Guo, Ying-Ying Hao, Xiao-Ming Wang, Hui-Na Zhang, Wan-Qiu Xiao, Nan Li, Jie Cong, Ying-Hao Han, Taeho Kwon

**Affiliations:** 1 Stem Cell and Regenerative Biology Laboratory, College of Life Science & Biotechnology, Heilongjiang Bayi Agricultural University, Xingyang Road #2, Daqing, 163319 Heilongjiang China; 2 Department of Biological Sciences, Research Center of Ecomimetics, Chonnam National University, 77 Yongbong-Ro, Buk-Gu, Gwangju, 61186 Republic of Korea; 3 National Coarse Cereals Engineering Research Center, Heilongjiang Bayi Agricultural University, Daqing, 163319 China; 4 Yabian Academy of Agricultural Science, Longjing, Jilin 1334000 China; 5 Primate Resources Center, Korea Research Institute of Bioscience and Biotechnology (KRIBB), 351-33 Neongme-Gil, Ibam-Myeon, Jeongeup-Si, Jeonbuk, 56216 Republic of Korea; 6 Department of Functional Genomics, KRIBB School of Bioscience, University of Science and Technology, Daejeon, 34113 Republic of Korea

**Keywords:** Pulmonary fibrosis, Peroxiredoxin 1, Reactive oxygen species, Epithelial-mesenchymal transition, Cell proliferation, PI3K/Akt and JNK/Smad signalling pathways

## Abstract

**Background:**

Pulmonary fibrosis is a major category of end-stage changes in lung diseases, characterized by lung epithelial cell damage, proliferation of fibroblasts, and accumulation of extracellular matrix. Peroxiredoxin 1 (PRDX1), a member of the peroxiredoxin protein family, participates in the regulation of the levels of reactive oxygen species in cells and various other physiological activities, as well as the occurrence and development of diseases by functioning as a chaperonin.

**Methods:**

Experimental methods including MTT assay, morphological observation of fibrosis, wound healing assay, fluorescence microscopy, flow cytometry, ELISA, western blot, transcriptome sequencing, and histopathological analysis were used in this study.

**Results:**

PRDX1 knockdown increased ROS levels in lung epithelial cells and promoted epithelial-mesenchymal transition (EMT) through the PI3K/Akt and JNK/Smad signalling pathways. PRDX1 knockout significantly increased TGF-β secretion, ROS production, and cell migration in primary lung fibroblasts. PRDX1 deficiency also increased cell proliferation, cell cycle circulation, and fibrosis progression through the PI3K/Akt and JNK/Smad signalling pathways. BLM treatment induced more severe pulmonary fibrosis in PRDX1-knockout mice, mainly through the PI3K/Akt and JNK/Smad signalling pathways.

**Conclusions:**

Our findings strongly suggest that PRDX1 is a key molecule in BLM-induced lung fibrosis progression and acts through modulating EMT and lung fibroblast proliferation; therefore, it may be a therapeutic target for the treatment of BLM-induced lung fibrosis.

**Supplementary Information:**

The online version contains supplementary material available at 10.1186/s11658-023-00460-x.

## Background

The peroxiredoxin (PRDX) family comprises ubiquitous peroxidases with a relative molecular weight of 22–27 kDa and includes six members in humans: PRDX1–6 [1]. PRDX1 is known to act as a scavenger of reactive oxygen species (ROS). It can inhibit oxidative stress-induced cellular damage [2] and interact with proteins as a molecular chaperone, exhibiting multiple functions in modulating cell proliferation, differentiation, apoptosis, ageing, cancer, and other diseases [3]. Changes in cellular redox status have been found to be associated numerous lung diseases, and PRDX1 expression is elevated in the lung tissues of patients with pulmonary fibrosis [4].

Pulmonary fibrosis is a chronic, irreversible, and fatal lung disease, which leads to respiratory dysfunction in humans. Patients suffer shortness of breath and a lack of oxygen, which can lead to lung failure and death due to asphyxia [5, 6]. The average life expectancy after diagnosis is 3–5 years, and there is no effective treatment available except lung transplantation [7]. Smoking and exposure to metal particles, cotton wool, and natural minerals are environmental risk factors inducing pulmonary fibrosis [8, 9]. In addition to environmental factors, lung injury induced by some drugs, such as amiodarone, paraquat, and bleomycin (BLM), is also of concern [10]. BLM is one of the main drugs used for modelling pulmonary fibrosis due to the lack of BLM hydrolase in the skin and especially in the lungs, which are highly sensitive to BLM [11].

The human lungs are vulnerable to oxidative stress [12], which is believed to be closely related to pulmonary fibrosis [13]. There is abundant oxygen and many mitochondria in the lung tissue; the mitochondria consume oxygen and produce ROS [14]. In addition, BLM produces large amounts of ROS when exposed to oxygen [15]. ROS can damage pulmonary epithelial cells [16] and stimulate epithelial cells to release high levels of cytokines, leading to epithelial-mesenchymal transition (EMT) [17], which is considered to be an important process in the development of pulmonary fibrosis. EMT is a biological process in which epithelial cells lose contact and adhesion, and obtain some mesenchymal characteristics of invasion, migration, and production of extracellular matrix through drastic changes in the shape of cytoskeleton [18].

TGF-β can reduce the activity of mitochondrial electron transfer chain in epithelial cells, leading to a loss of mitochondrial transmembrane potential and an increase in mitochondrial ROS production. Simultaneously, high levels of ROS in the epithelial cells can induce the oxidation and activation of TGF-β to promote fibroblast aggregation [19–22]. Repeated injury of epithelial cells activates the wound healing mechanisms, promoting cytokine release, which stimulates the proliferation of fibroblasts and increases the expression of collagen [6, 23, 24]. Concurrently, The TGF-β1 signalling pathway is the main cascade reaction of fibroblast differentiation [8]. TGF-β signalling induces many events, including an increase in the number of cells responsible for collagen production and matrix deposition, the production of extracellular matrix, and the expression of α-smooth muscle actin (α-SMA) [8, 25, 26], further accelerating the process of pulmonary fibrosis. However, further exploration is needed to determine whether PRDX1 can regulate fibroblast fibrosis to affect the development of pulmonary fibrosis.

The phosphoinositide 3-kinase (PI3K)/protein kinase B (AKT) signalling pathway is one of the central signalling pathways that regulate cellular activities [27]. PI3Ks are a group of lipid kinases associated with the plasma membrane, which can be expressed in human lung fibroblasts [28]. BLM-induced pulmonary fibrosis relies on the production of ROS, which can participate in lung injury through the PI3K/AKT signalling pathway. PI3K is overexpressed in pulmonary fibrosis lung homogenates and fibroblasts [29], indicating that PI3K may have an impact on pulmonary fibrosis [30]. AKT activation can induce hydrogen peroxide release and damage adjacent lung epithelial cells, thereby promoting pulmonary fibrosis [31]. AKT-deficient mice exhibit resistance against BLM-induced pulmonary fibrosis and inflammation [32]. JNKs are stress-activated protein kinases that can be induced by various stimuli and play an important role in the pathological mechanisms of fibrosis, including epithelial cell damage, fibroblast activation, and collagen production [33]. JNK1 has been shown to have anti-fibrotic effects, indicating that JNK1 inhibition may be an important therapeutic target for pulmonary fibrosis [34]. TGF-β1 and TGF-β3 can directly stimulate the activation of JNK through phosphorylation-dependent mechanisms [35]. JNK is also involved in the phosphorylation of Smad2/3 and is essential for TGF-β-induced transcriptional activation [36]. However, it is currently not clear whether PRDX1 regulates the proliferation ability of fibroblasts through the JNK/Smad pathway, thereby affecting the development of pulmonary fibrosis.

In the process of BLM-induced pulmonary fibrosis, *PRDX1*-knockout (KO) mice produce more severe pulmonary inflammation and pulmonary fibrosis [37]. Therefore, PRDX1 is of great interest for the research on the occurrence and development of pulmonary fibrosis. Although it has been found that PRDX1 has an impact on the development of pulmonary fibrosis, the role of PRDX1 in BLM-induced pulmonary fibrosis remains elusive. Therefore, in this study, we further explored the role of PRDX1 in BLM-induced pulmonary fibrosis. PRDX1 knockdown in pulmonary epithelial cells increases ROS production and mitochondrial damage, accelerates EMT, increases TGF-β secretion, and induces the activation of the JNK/Smad signalling pathway. In pulmonary fibroblasts, PRDX1 knockdown significantly increases cellular proliferation and induces the secretion of collagen by promoting the activation of the PI3K/AKT and the JNK/Smad signalling pathways, further exacerbating pulmonary fibrosis. BLM induces more severe pulmonary fibrosis in PRDX1-KO mice as well as a strong activation of PI3K/AKT and JNK/Smad signalling pathways.

## Materials and methods

### Materials

The lung epithelial cell line BEAS-2B (iCell-h023) was purchased from Daqing Hongtu Biotechnology Co., Ltd. (Heilongjiang, China).

All experimental procedures involving the use of animals were approved by the Heilongjiang Bayi Agricultural University Animal Ethics Committee. The mice were housed in a clean animal facility at Heilongjiang Bayi Agricultural University under a 12:12 h light/dark cycle and fed normal mouse food. Mice were used for natural mating and breeding at 6–8 months of age.

Phosphate-buffered saline (PBS; SH30256.01B) and Dulbecco’s modified Eagle’s medium (DMEM; SH30243.01) were obtained from Cytiva (Marlborough, MA, USA); dihydroethidium (DHE; ID3560), mitochondrial superoxide (Mito-SOX; M36008), JC-1 (M8650), and Hoechst (C0031) reagents from Solarbio Science and Technology Co., Ltd. (Beijing, China); propidium iodide (PI; C1052) from Beyotime Biotechnology Co., Ltd. (Nanjing, China); penicillin/streptomycin (P/S; P7630), trypsin/EDTA solution (TE; T1300), and foetal bovine serum (FBS; #11011-8611) from Solarbio Science and Technology Co., Ltd. (Beijing, China); 3-(4,5-dimethylthiazol-2-yl)-2,5-diphenyltetrazolium bromide (MTT; #298-93-1) from Amresco, VWR Life Science (Radnor, PA, USA); dimethyl sulfoxide (DMSO; D1435) from Sigma-Aldrich (Merck KGaA, Darmstadt, Germany); BLM (R25001) from Thermo Fisher Scientific, Inc. (Waltham, MA, USA); the antibodies for β-actin (sc-4778), N-cadherin (sc-8424), E-cadherin (sc-7870), vimentin (c-7870), fibronectin (sc-8422), and slug (sc-166476) from Santa Cruz Biotechnology, Inc. (Dallas, TX, USA); TGF-β1 enzyme-linked immunosorbent assay kit (ELISA; DB100C) from R&D Systems, Inc. (Minneapolis, MN, USA); and Cell Cycle Analysis Kit (C1052) from Beyotime Biotechnology (Shanghai, China).

### Isolation of foetal mouse lung fibroblast cells

The wild-type (WT) and *PRDX1*-KO 129/SvJ female mice (Korea Research Institute of Bioscience and Biotechnology, Daejeon, Korea) were euthanised humanely using CO_2_ on the 20th day of pregnancy. Foetuses were harvested, and the foetal lungs were removed, minced, incubated with 0.25% TE for 20 min at 37 °C, and filtered through 70-μm filters. WT and PRDX1-KO primary foetal mouse lung fibroblasts (FMLF) were isolated and purified by differential attachment, inoculated in DMEM medium (containing 10% FBS, 1% P/S), and cultured in an incubator (37 °C, 5% CO_2_).

### Lentivirus transfection

The lentiviral vectors shPRDX1-LV2 (U6 & puro) and Mock-LV2 (U6 & puro) were purchased from Shanghai Gene Pharma Co., Ltd. (Shanghai, China). The BEAS-2B cells were seeded in a 6-well culture plate at a density of 2 × 10^5^ cells per well. After stably adhering to the wall, the cells were incubated with lentivirus for 72 h. The transfected cells were screened using puromycin. After successful transfection and amplification, the cells were frozen and stored for future use. PRDX1 protein levels were examined by western blotting to confirm the knockdown. Each experiment was repeated three times.

### MTT assay

To assess cell viability, the cells were inoculated in a 96-well culture plate (5 × 10^3^ cells/well) in DMEM supplemented with 1% FBS and 1% P/S. The cells were then treated with BLM at indicated concentrations for 72 h and incubated with MTT for 4 h. The supernatant was discarded, and DMSO was added. After 10 min, the absorbance was measured at 570 nm. Each experiment was repeated three times.

To assess cell proliferation, the cells were inoculated in a 96-well culture plate (5 × 10^3^ cells/well) in DMEM supplemented with 10% FBS and 1% P/S. The cells were treated with BLM as indicated, followed by incubation with MTT for 4 h. Then, the supernatant was discarded, and DMSO was added to each well. After 10 min, the absorbance was measured at 570 nm. Each experiment was repeated three times.

### Fibrosis morphological observation

The cells were inoculated in a 24-well culture plate (6 × 10^4^ cells/well). After adhesion, the cells were treated with BLM as indicated and observed using an inverted microscope. Each experiment was repeated three times.

### Wound healing assay

The cells were inoculated in a 24-well culture plate (2 × 10^5^ cells/well). After adhesion, the cells were vertically scribed and washed with PBS three times. The cells were then cultivated in serum-free culture medium and incubated with BLM (10 μg/mL for 72 h). The images were acquired at 0 h and 72 h. Image J software (National Institutes of Health, Bethesda, MD, USA) was used to calculate the scratch area. Healing ratio was calculated using the following formula: Area healing (%) = 100 × (average area at time 0 h − average area at time 72 h) / average area at time 0 h. Each experiment was repeated three times.

### Fluorescence microscopy

*DHE/Hoechst staining:* The cells were inoculated in a 24-well culture plate (6 × 10^4^ cells/well). After the cells were treated with BLM (10 μg/mL for 72 h), the supernatant was discarded, and the cells were washed twice with PBS. Then, DHE (5 μM) and Hoechst (1 μg/mL) dyes were added, and the cells were incubated in a cell culture incubator for 20 min, after which the dye solution was discarded, and the cells were washed once with PBS. PBS was added, and the cells were imaged using a fluorescence microscope. Each experiment was repeated three times.

*Mito-SOX/Hoechst staining:* The cells were inoculated in a 24-well culture plate (6 × 10^4^ cells/well). After the cells were treated with BLM as indicated, the supernatant was discarded, and the cells were washed twice with PBS. The cells were then fixed with 4% paraformaldehyde solution. Mito-SOX (5 μM) and Hoechst (1 μg/mL) dyes were added, and the cells were incubated in a cell culture incubator for 20 min. The dye solution was discarded, and the cells were washed once with PBS. Afterwards, PBS was added, and the cells were imaged using a fluorescence microscope. Each experiment was repeated three times.

*JC-1 staining:* The cells were inoculated in a 24-well culture plate (6 × 10^4^ cells/well). After the cells were treated with BLM (10 μg/mL for 72 h), the supernatant was discarded, and the cells were washed twice with PBS. Next, JC-1 (10 μg/mL) dye was added, and the cells were incubated in a cell culture incubator for 20 min. The dye solution was discarded, and the cells were washed once with PBS. After adding PBS, the cells were imaged using a fluorescence microscope. Each experiment was repeated three times.

### Flow cytometry

*DHE staining:* The cells were inoculated in a 6-well culture plate (2 × 10^5^ cells/well). After the cells were treated with BLM (10 μg/mL for 72 h), the supernatant was discarded, and the cells were washed once with PBS. Next, the cells were collected in TE, transferred into a tube, and washed once with PBS. Subsequently, DHE (5 μM) dye was added, and the cells were incubated in a cell culture incubator for 20 min. The dye solution was discarded, and the cells were washed once with PBS and resuspended in PBS. Subsequently, flow cytometry was performed. Each experiment was repeated three times.

*Propidium iodide (PI) staining:* The cells were inoculated in a 6-well culture plate (2 × 10^5^ cells/well). After the cells were treated with BLM (10 μg/mL for 72 h), the supernatant was discarded. The cells were washed once with PBS, collected in TE, and transferred into a tube. The collected cells were washed once with PBS and fixed with 70% ethanol at 4 °C for 12 h. Afterwards, the cells were centrifuged, and the supernatant was discarded before washing them once with PBS. Then, PI (50 μg/mL) dye was added, and the cells were incubated in a cell culture incubator for 30 min. The dye solution was discarded, and the cells were washed once with PBS and resuspended in PBS. Subsequently, flow cytometry was performed. Each experiment was repeated three times.

### ELISA

*Cell supernatant:* The cells were inoculated in a 6-well culture plate (2 × 10^5^ cells/well). After the cells were treated with BLM (10 μg/mL for 72 h), the supernatant was removed. A TGF-β1 ELISA kit was used to detect the content of TGF-β1 secreted by the cells. Each experiment was repeated three times.

*Mouse serum:* Pulmonary fibrosis was induced in 8-week-old WT and *PRDX1-*KO 129/SvJ mice by intratracheal administration of BLM (5 mg/kgBW in 50 μL PBS, single administration; N = 6). The control group received the same volume of sterile PBS (N = 6). After 21 days, the mice were subjected to eyeball blood collection, and the blood samples were left to stand before centrifugation to extract serum. A TGF-β1 ELISA kit was used to determine TGF-β1 levels in mouse serum. Each experiment was repeated three times.

### Western blot

*Cells:* The cells were inoculated in a 6-well culture plate (2 × 10^5^ cells/well). After the cells were treated with BLM as indicated, they were collected and lysed in protein lysis buffer (20 mM HEPES-OH, pH 7.0; 50 mM NaCl; 10% glycerol; 0.5% Triton X 100) for total protein extraction. The extracted protein samples were boiled for 5 min, separated using 12% sodium dodecyl sulfate–polyacrylamide gel electrophoresis, and transferred onto nitrocellulose membranes. The membranes were blocked in 5% skimmed milk for 30 min at room temperature and incubated with the primary antibodies polyclonal rabbit anti-N-cadherin, mouse monoclonal anti-E-cadherin, anti-vimentin, anti-fibronectin, anti-slug, and anti-β-actin (dilution, 1:1000) at 4 °C overnight. The membranes were washed five times with tris-buffered saline containing Tween (TBST; 150 mM NaCl, 10 mM Tris HCl [pH 7.5], and 0.2% Tween-20). Subsequently, the membranes were incubated with horseradish peroxidase-conjugated goat anti-mouse IgG or anti-rabbit IgG for 1 h at room temperature. After removing the excess antibodies by washing with TBST, specific conjugates were detected using a chemiluminescence detection system according to the manufacturer’s protocol. Each experiment was repeated three times.

*Lung tissues:* Pulmonary fibrosis was induced in 8-week-old WT and *PRDX1-*KO 129/SvJ mice by intratracheal administration of BLM (5 mg/kgBW in 50 μL PBS, single administration; N = 6). The control group received the same volume of sterile PBS (N = 6). The mice were sacrificed 21 days after the BLM injection, and the lungs were collected for analysis. The detection of fibrosis related protein expression in lung tissue was performed as described above for cell culture. Each experiment was repeated three times.

### Transcriptome sequencing analysis

After the cells were treated with BLM (10 μg/mL for 72 h), an appropriate amount of Trizol was added according to the cell volume to lyse the cells. The samples were sent to the Nanjing Pineson Gene Technology Co., Ltd. (Nanjing, China) for transcriptome sequencing analysis. Each experiment was repeated three times.

### Histopathological analysis

Pulmonary fibrosis was induced in 8-week-old WT and *PRDX1-*KO 129/SvJ mice by intratracheal administration of BLM (5 mg/kgBW in 50 μL PBS, single administration; N = 6). The control group received the same volume of sterile PBS (N = 6). Body temperature and weight of the mice were recorded after BLM treatment. The mice were sacrificed 21 days after the BLM injection, and the lungs were collected for analysis. Pathological sections of the tissue were prepared and stained with hematoxylin and eosin (H&E). Neutral resin sealing was performed, and the samples were imaged under a microscope. Each experiment was repeated three times.

### Statistical analysis

Repeated measures analysis of variance was used to analyse changes in time and differences between groups in each experiment. Data were analysed using an independent-samples t-test. Differences with p-values less than 0.05 were considered statistically significant.

## Results

### PRDX1 knockdown promoted EMT and increased ROS levels in lung epithelial cells

**Fig. 1:**
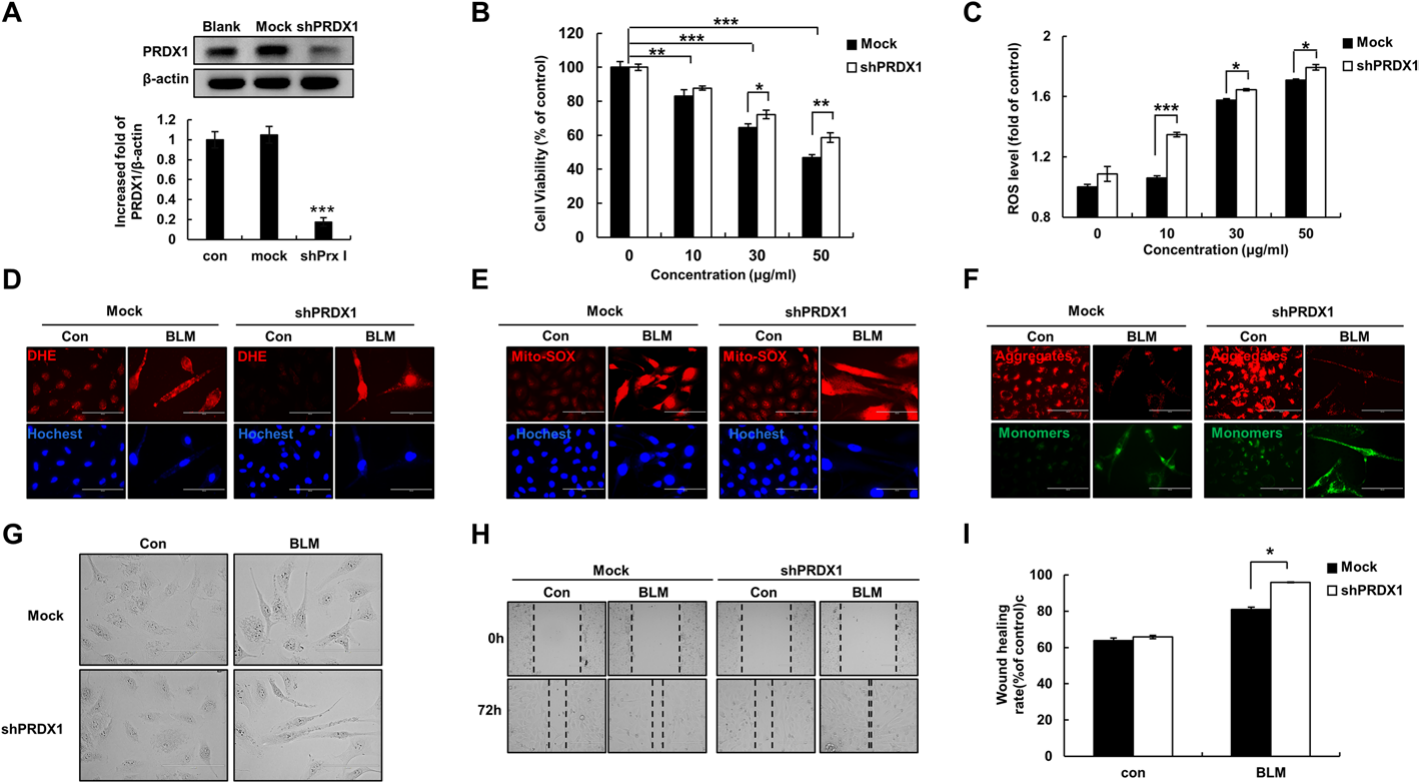
Effect of PRDX1 on BLM-induced viability, ROS levels, and epithelial cell migration. Besa-2B cells were treated with 10 μg/mL BLM for 72 h (panels D–I). **A** PRDX1 protein levels were examined by western blot. **B** Cell viability was estimated by MTT assay. **C** The level of intracellular ROS was estimated by flow cytometry. **D** The levels of intracellular ROS was monitored by DHE fluorescence photography. **E** The levels of mitochondrial ROS were measured using Mito-SOX. **F** Mitochondrial membrane potential was determined using JC-1. **G** The morphology of cellular fibrosis was observed under microscope. **H** Cell migration was estimated by wound healing assay. **I** Quantitative analysis of data presented in panel H. *, *p* < 0.05; **, *p* < 0.01; ***, *p* < 0.001. *PRDX1* peroxiredoxin, *BLM* bleomycin, *ROS* reactive oxygen species, *MTT* 3-(4,5-dimethylthiazol-2-yl)-2,5-diphenyltetrazolium bromide, *DHE* dihydroethidium, *Mito-SOX* mitochondrial superoxide

The mock and shPRDX1 cells were constructed by transfecting BESA-2B cells with Mock-LV2 and shPRDX1-LV2 lentiviruses, respectively. Western blot analysis showed that the level of PRDX1 protein in shPRDX1 cells was significantly reduced, indicating that PRDX1-knockdown cells were successfully constructed (Fig. 1A). The cells were then treated with BLM (0, 10, 30, or 50 μg/mL) for 72 h. The viability of cells, as determined by MTT assay, significantly decreased with increasing BLM concentration, and the viability of shPRDX1 cells was higher than that of mock cells (Fig. 1B). Quantitative flow cytometry analysis showed that the intracellular ROS levels of shPRDX1 cells were significantly higher than those of the mock cells after treatment with 10 μg/mL BLM (Fig. 1C). Fluorescence images showed that, compared with the mock cells, intracellular (Fig. 1D) and mitochondrial (Fig. 1E) ROS levels in shPRDX1 cells were increased; in contrast, mitochondrial membrane potential (Fig. 1F) was decreased. After 10 μg/mL BLM treatment for 72 h, noticeable fibrotic changes were observed in cell morphology (Fig. 1G). The wound healing assay showed that the migration ability of shPRDX1 cells was significantly higher than that of the mock cells (Fig. 1H). These results indicated that PRDX1 knockdown significantly promoted mitochondrial damage caused by BLM-induced oxidative stress in epithelial cells. Furthermore, PRDX1 knockdown induced morphological changes in epithelial cells and enhanced cell migration ability.

### PRDX1 knockdown promoted the epithelial EMT process through the PI3K/Akt and JNK/Smad signalling pathways

**Fig. 2:**
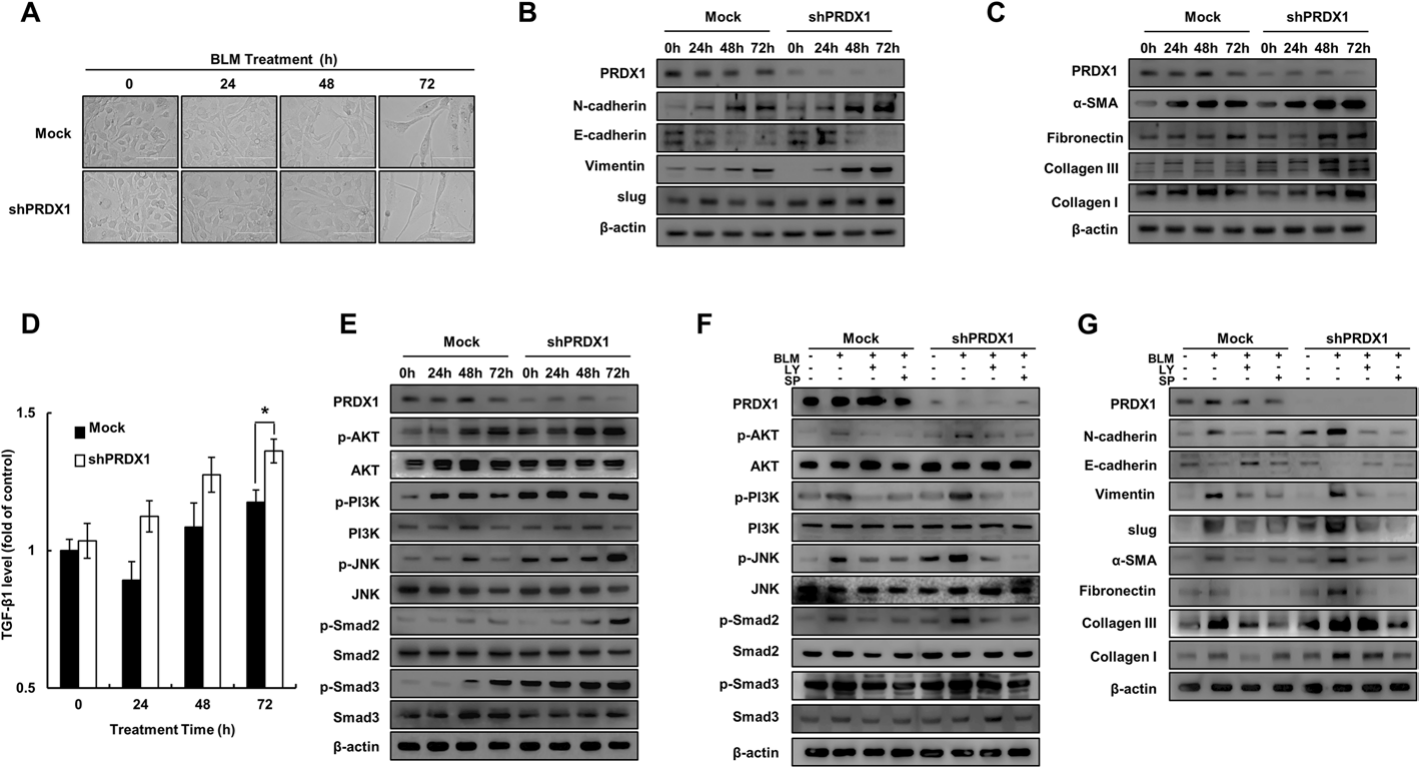
Effect of PRDX1 on BLM-induced EMT and the PI3K/Akt and JNK/Smad signalling pathways in epithelial cells. The BEAS-2B cells were treated with 10 μg/mL BLM. **A** The morphology of cellular fibrosis was observed under a microscope. **B** The expression levels of EMT-related proteins. **C** The expression levels of fibrosis-related proteins. **D** Secreted TGF-β1 levels as determined using an ELISA kit. **E** The expression levels of the PI3K/Akt and JNK/Smad signalling pathway-related proteins. **F** The expression levels of the PI3K/Akt and JNK/Smad signalling pathway-related proteins. **G** The expression levels of EMT and fibrosis-related proteins. Protein levels were determined by western blot analysis. *, *p* < 0.05; **, *p* < 0.01; ***, *p* < 0.001. *PRDX1* peroxiredoxin, *BLM* bleomycin, *EMT* epithelial-mesenchymal transition, *PI3K* phosphoinositide 3-kinase, *Akt* protein kinase B, *JNK* c-Jun N-terminal kinase, *Smad* small of mothers against decapentaplegic, *TGF-β1* transforming growth factor β1, *ELISA* enzyme-linked immunosorbent assay

Pulmonary fibrosis is a progressive, irreversible, and ultimately fatal chronic lung disease. The progress of the disease is generally slow [38]. To better observe the occurrence and development of pulmonary fibrosis disease, we observed the cell morphology after 24, 48, and 72 h of BLM treatment (30 μg/mL). The cells exhibited a clear fibrosis transformation at 72 h (Fig. 2A and Additional file 1: Fig. S1A). PRDX1 knockdown also significantly increased the expression levels of EMT-associated proteins (Fig. 2B and Additional file 1: Fig. S1B) and fibrosis-related proteins (Fig. 2C). PRDX1 knockdown promoted the TGF-β1 secretion at 72 h (Fig. 2D; measured by ELISA). PI3K/Akt and JNK/Smad protein phosphorylation levels were significantly elevated after 48 h and 72 h of BLM treatment (Fig. 2E and Additional file 1: Fig. S2A), indicating that these signalling pathways were activated. Pre-treatment with the inhibitors of PI3K (SP600125) and JNK (LY294002) restored the activity of these signalling pathways (Fig. 2F and Additional file 1: Fig. S2B) and the expression levels of EMT-associated and fibrosis-related proteins (Fig. 2G and Additional file 1: Fig. S3). These results indicated that PRDX1 knockdown significantly promoted the EMT of epithelial cells and collagen expression by strongly activating the JNK–Smad signalling pathway. The promotion of EMT can also explain the morphological changes observed in epithelial cells.

### PRDX1 knockout significantly increased TGF-β secretion, ROS production, and cell migration in primary lung fibroblasts

**Fig. 3:**
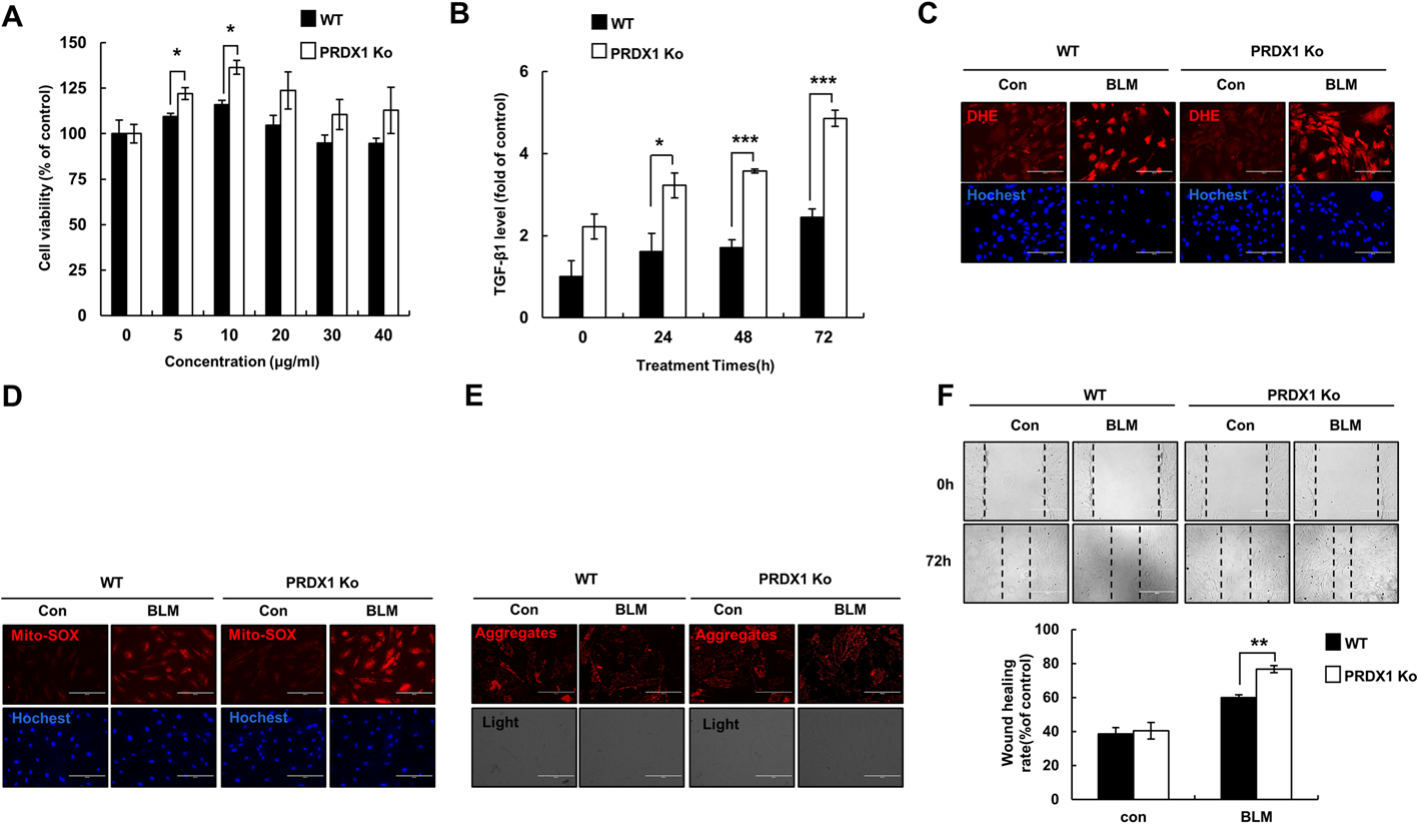
Effect of PRDX1 on TGF-β secretion and oxidative stress in primary pulmonary fibroblasts. FMLF were treated with 10 μg/mL BLM for 72 h (panels C–F). **A** Cell viability was estimated by MTT assay. **B** TGF-β1 secreted by the cells was quantified using an ELISA kit. **C** The levels of intracellular ROS were measured using DHE. **D** The ROS levels in the mitochondria were determined using Mito-SOX. **E** The mitochondrial membrane potential was measured using JC-1. **F** Cell migration was estimated by wound healing assay. *, *p* < 0.05; **, *p* < 0.01; ***, *p* < 0.001. *PRDX1* peroxiredoxin, *TGF-β1* transforming growth factor β1, *FMLF* foetal mouse lung fibroblasts, *BLM* bleomycin, *MTT* 3-(4,5-dimethylthiazol-2-yl)-2,5-diphenyltetrazolium bromide, *TGF-β1* transforming growth factor β1, *ELISA* enzyme-linked immunosorbent assay, *ROS* reactive oxygen species, *DHE* dihydroethidium, *Mito-SOX* mitochondrial superoxide

Epithelial cell damage triggers a self-repair mechanism. With repeated damage and insufficient repair, fibroblasts proliferate excessively to replace the epithelial cells. As a result, pulmonary function is gradually impaired, and pulmonary fibrosis is aggravated [39]. Primary mouse lung fibroblasts were treated with BLM (0, 5, 10, 20, 30, 40 μg/mL) for 72 h. The MTT assay showed that the viability of PRDX1-KO cells was significantly higher than that of WT cells after exposure to 10 μg/mL BLM (Fig. 3A). Furthermore, TGF-β1 secretion by PRDX1-KO cells was significantly higher than that by the WT cells at 72 h (Fig. 3B). Intracellular and mitochondrial ROS levels in PRDX1-KO cells were significantly elevated compared to control cells (Fig. 3C, D); however, there was no significant difference in ROS levels between the two groups (Fig. 3E). The migration ability of PRDX1-KO cells was significantly higher than that of WT cells (Fig. 3F).

These results demonstrate that PRDX1 deficiency can promote cell viability and TGF-β1 secretion. Moreover, BLM treatment can significantly enhance the migration ability of primary lung fibroblasts, which further implies a change in fibroblast proliferation and differentiation ability [40]. There were no obvious differences in mitochondrial damage, possibly because lung fibroblasts are not sensitive to ROS at the late stage of BLM treatment.

### PRDX1 knockout increased cell proliferation, cell cycle circulation, and fibrosis progression through the PI3K/Akt and JNK/Smad signalling pathways

**Fig. 4:**
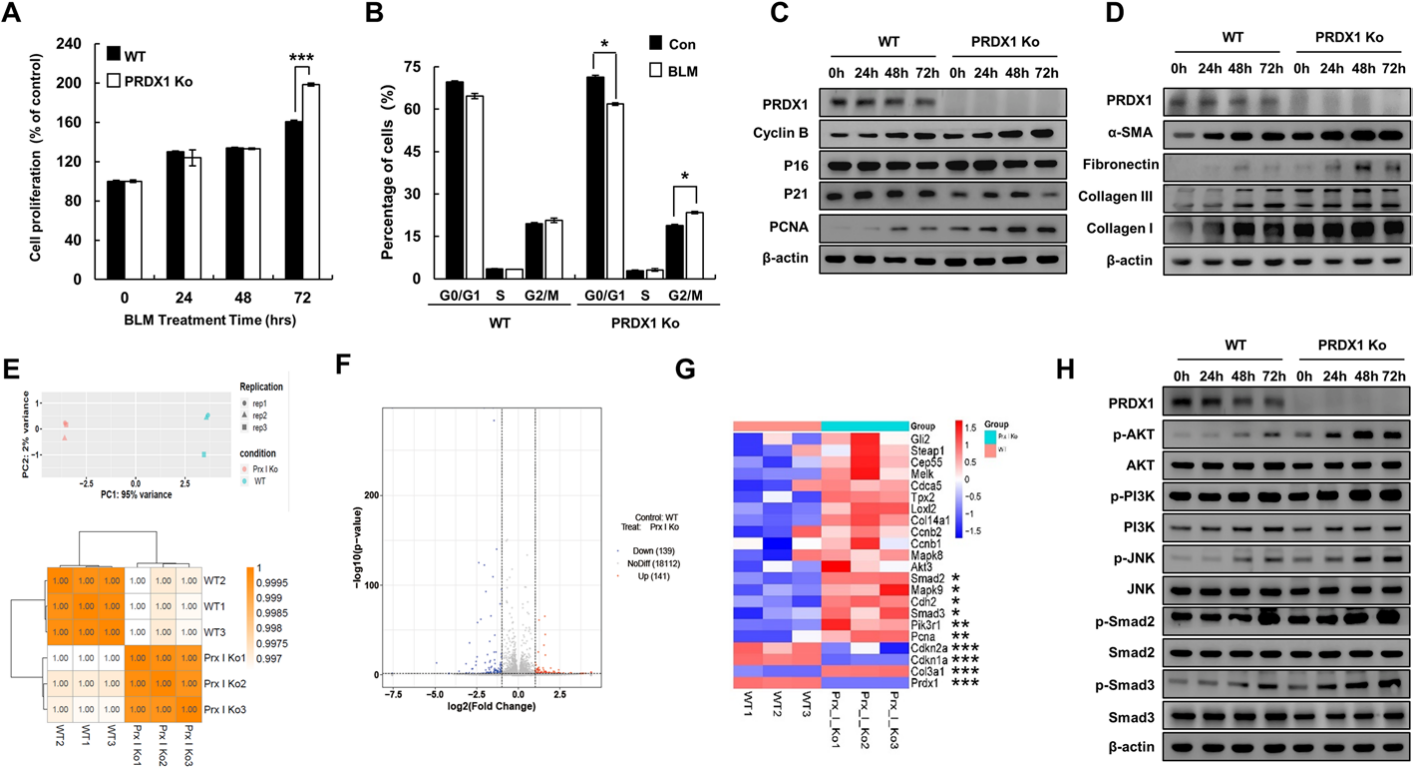
Effects of PRDX1 on proliferation, collagen-associated protein expression, and the PI3K/Akt and JNK/Smad signalling pathways in primary pulmonary fibroblasts. FMLF were treated with 10 μg/mL BLM. **A** Cell viability was estimated by MTT assay. **B** The cell cycle stage was estimated by flow cytometry. **C** The expression levels of cell cycle-related proteins. **D** The expression levels of fibrosis-related proteins. **E** Sample correlation was analysed by Pearson correlation coefficient and principal components analysis. **F** Differentially expressed genes in volcano mapping. **G** Cluster analysis of differentially expressed genes. **H** The expression levels of PI3K/Akt and JNK/Smad signalling pathways-related proteins. Protein levels were determined by western blot analysis. *, *p* < 0.05; **, *p* < 0.01; ***, *p* < 0.001. *PRDX1* peroxiredoxin, *PI3K* phosphoinositide 3-kinase, *Akt* protein kinase B, *JNK* c-Jun N-terminal kinase, *Smad* small of mothers against decapentaplegic, *FMLF* foetal mouse lung fibroblasts, *BLM* bleomycin, *MTT* 3-(4,5-dimethylthiazol-2-yl)-2,5-diphenyltetrazolium bromide

The proliferation of pulmonary fibroblasts is an important part of pulmonary fibrosis [41]. The cells were treated with 10 μg/mL of BLM for 0, 24, 48, and 72 h. The MTT assay showed that the activity of PRDX1-KO cells decreased significantly; furthermore, the viability of PRDX1-KO cells was higher than that of the WT cells (Fig. 4A). Compared with the WT cells, PRDX1-KO cells in the G0/G1 phase increased in numbers, and those in the S and G2/M phases decreased (Fig. 4B). In addition, the proliferation capacity of PRDX1-KO cells increased significantly. Western blot analyses showed that PRDX1 deficiency significantly promoted cell cycle progression (Fig. 4C and Additional file 1: Fig. S4A) and collagen expression (Fig. 4D and Additional file 1: Fig. 4B).

After BLM treatment, transcriptome sequencing was performed on WT and PRDX1-KO FMLF cells to analyse differential gene expression. In PRDX1-KO cells, Cyclin *P21* and *P16* were significantly down-regulated, collagen-related genes were significantly up-regulated, and *PI3K*, *JNK,* and *Smad* were up-regulated (Fig. 4E–G). Western blot analyses showed that PRDX1 knockout promoted the phosphorylation of PI3K, JNK, and Smad (Fig. 4H and Additional file 1: Fig. S4C). These results indicate that PRDX1 deficiency promoted S and G2/M cell cycle phases by strongly activating the JNK/Smad pathway, thereby accelerating the proliferation of the FMLF cells, promoting their transformation to myofibroblasts, increasing the expression of collagen, and finally promoting pulmonary fibrosis.

### PI3K/Akt and JNK/Smad signalling were the key pathways in BLM-induced pulmonary fibrosis in PRDX1-KO cells

**Fig. 5:**
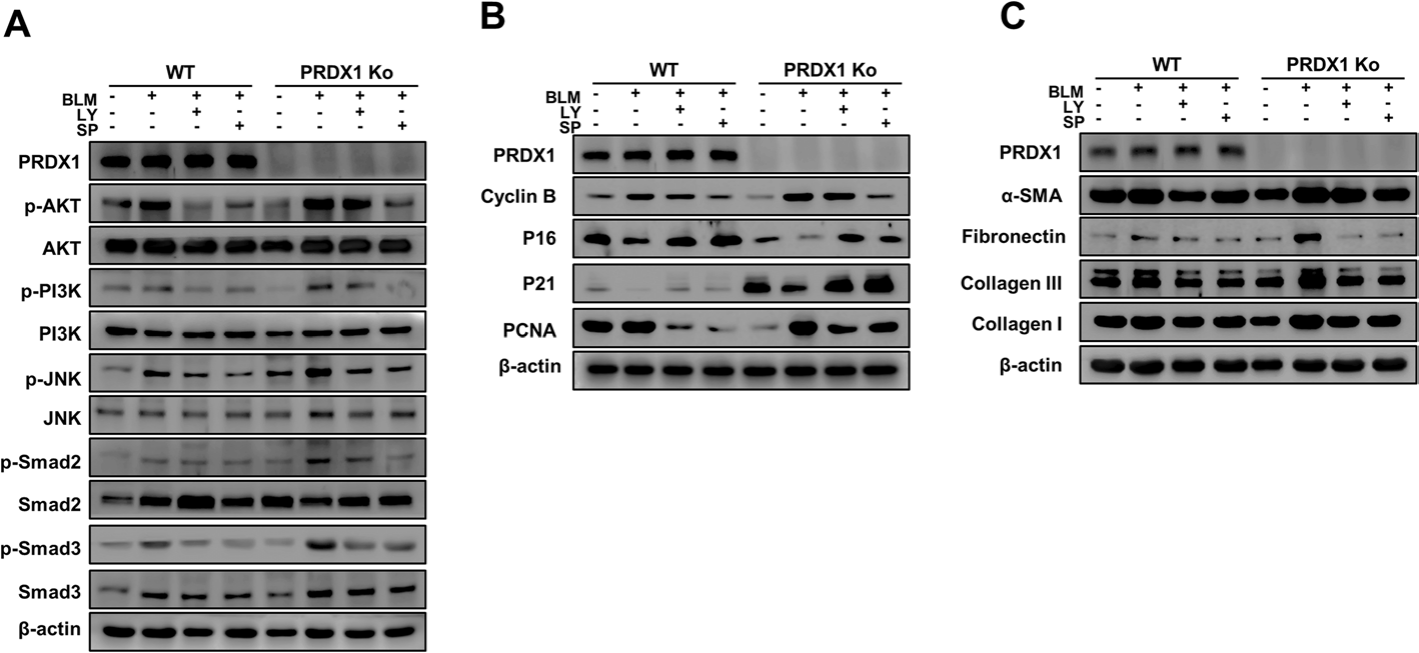
Effect of PI3K and JNK inhibitors on BLM-induced expression of proteins related to cell cycle and fibrosis in primary lung fibroblasts. FMLF were treated with 10 μg/mL BLM for 72 h. **A** The expression levels of PI3K/Akt and JNK/Smad signalling pathways-related proteins. **B** The expression levels of cell cycle-related proteins. **C** The expression levels of fibrosis-related proteins. Protein expression levels were determined by western blot analysis. *, *p* < 0.05; **, *p* < 0.01; ***, *P* < 0.001. *PI3K* phosphoinositide 3-kinase, *JNK* c-Jun N-terminal kinase, *BLM* bleomycin, *FMLF* foetal mouse lung fibroblasts, *Akt* protein kinase B, *Smad* small of mothers against decapentaplegic

To confirm the roles of the PI3K/Akt and JNK/Smad signalling pathways in PRDX1-associated pulmonary fibrosis regulation, FMLF cells were pre-treated with the inhibitors of PI3K (LY294002) and JNK (SP600125) (hereafter, SP and LY, respectively). Western blot analyses showed that the activation of the BLM-induced signalling pathways were significantly inhibited, and the levels of proteins related to EMT and fibrosis were altered in FMLF cells pre-treated with SP and LY (Fig. 5A and Additional file 1: Fig. S5A). SP had a notable inhibitory effect on the cell cycle (Fig. 5B and Additional file 1: Fig. S5B), while LY had a pronounced inhibitory effect on fibrosis-related proteins (Fig. 5C and Additional file 1: Fig. S5C).

### BLM induced more severe pulmonary fibrosis in *PRDX1*-KO mice

**Fig. 6:**
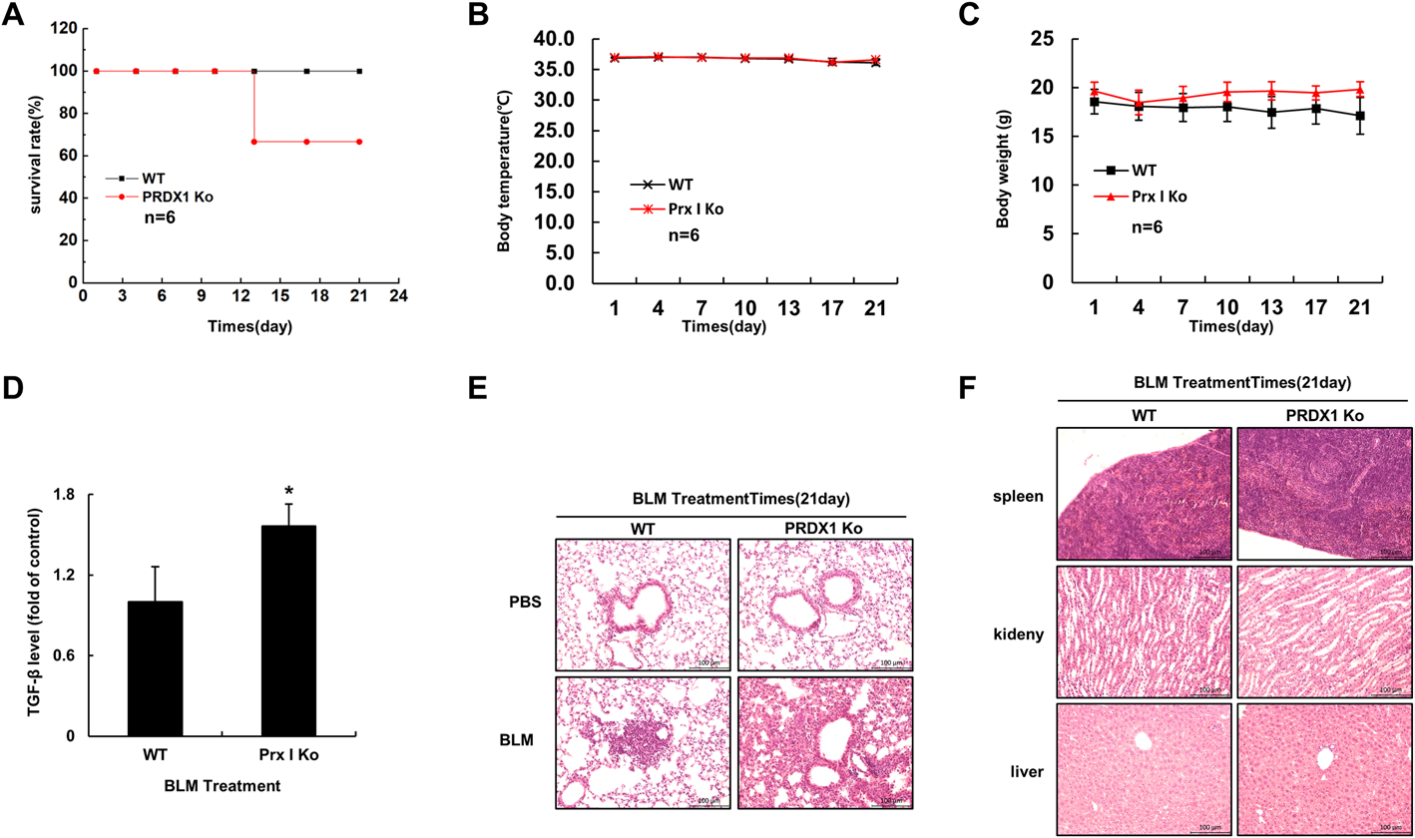
Effect of BLM on lethality and pulmonary fibrosis in mice. Lung fibrosis was induced in mice by intratracheal administration of BLM (5 mg/kgBW in 50 μL PBS) for 21 days. **A** Analysis of mouse mortality. **B** Mouse body temperature. **C** Body weight of mice. **D** Serum TGF-β1 levels in mice were estimated by ELISA. **E** Degree of pulmonary fibrosis in mice as determined by H&E staining. **F** The kidney, liver, and spleen samples of mice analysed by H&E staining. *, *p* < 0.05; **, *p* < 0.01; ***, *p* < 0.001. *BLM* bleomycin, *PBS* phosphate-buffered saline, *TGF-β1* transforming growth factor β1, *ELISA* enzyme-linked immunosorbent assay, *H&E* haematoxylin and eosin

To further confirm the regulatory role of PRDX1 in pulmonary fibrosis, BLM was injected into the lungs of mice to generate a pulmonary fibrosis model in vivo. During the second week after the BLM treatment, two *PRDX1*-KO mice died (Fig. 6A). The body temperature (Fig. 6B) and weight (Fig. 6C) data of the remaining mice were recorded longitudinally, revealing no significant fluctuation. The mice were euthanized on day 21 after the BLM treatment, and the serum TGF-β1 levels were determined by ELISA. The serum TGF-β1 levels of the *PRDX1*-KO mice were elevated (Fig. 6D). Moreover, as examined by H&E staining, the lungs of *PRDX1*-KO mice produced more severe pulmonary fibrosis (Fig. 6E); the other organs exhibited no significant changes (Fig. 6F).

**Fig. 7:**
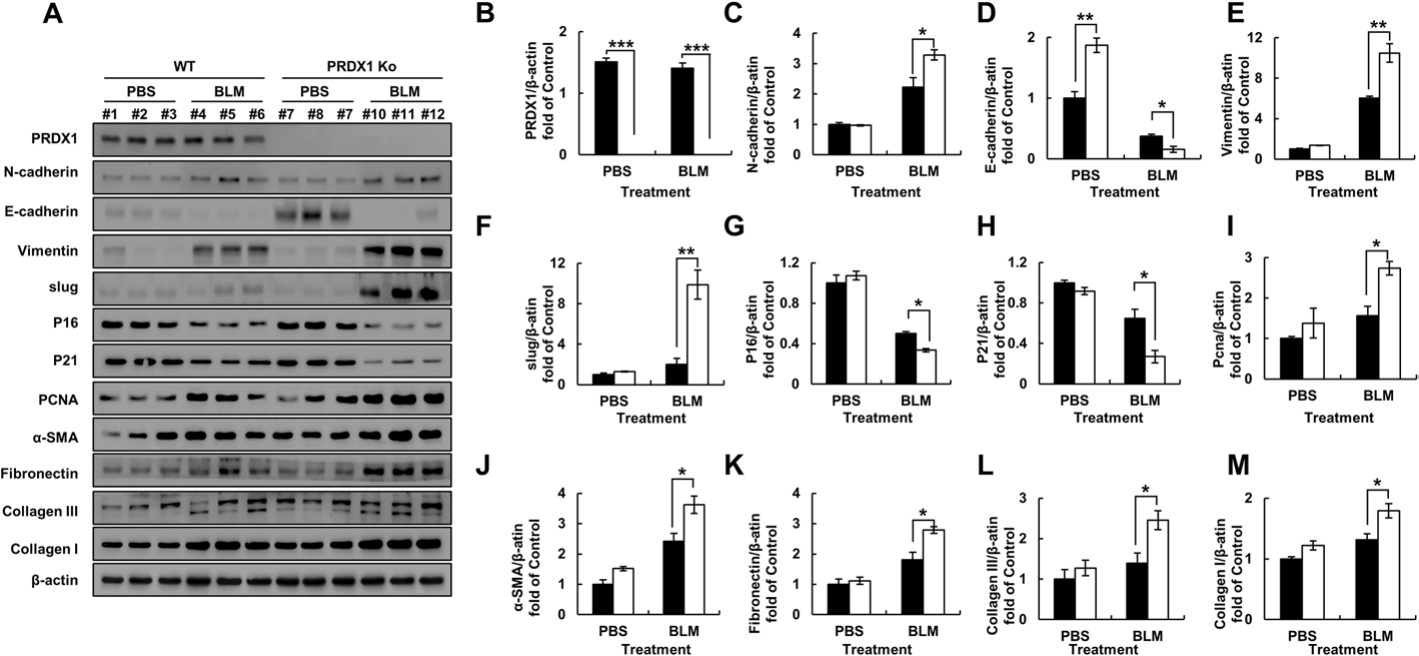
Expression of EMT and fibrosis-related proteins after BLM treatment in mice. Lung fibrosis was induced in mice by intratracheal administration of BLM (5 mg/kgBW in 50 μL PBS) for 21 days. **A** Expression of EMT (N-cadherin, E-cadherin, vimentin, slug), collagen-related (α-SMA, fibronectin, collagen I/III), cell cycle (P16, P21), and PCNA proteins in mouse lung tissues as measured by western blotting. **B**–**M** Quantitative analyses of the data presented in panel A. *, *p* < 0.05; **, *p* < 0.01; ***, *p* < 0.001. *EMT* epithelial-mesenchymal transition, *BLM* bleomycin, *PBS* phosphate-buffered saline, *α-SMA* α-smooth muscle actin, *PCNA* proliferating cell nuclear antigen

Further, to assess the molecular mechanism underlying the role of PRDX1 in BLM-induced pulmonary fibrosis in mice, the expression levels of fibrosis-related proteins were determined by western blot analysis. The levels of EMT-related proteins in the lung cells were found to be altered; cyclin P21 and P16 were down-regulated, while proliferating cell nuclear antigen protein, α-SMA, and collagen were up-regulated (Fig. 7A–M). These results indicate that PRDX1 can promote pulmonary fibrosis in mice by regulating EMT and cyclin, as well as collagen protein expression.

### PRDX1 promotes BLM-induced pulmonary fibrosis in mice through the PI3K/Akt and JNK/Smad signalling pathways

**Fig. 8:**
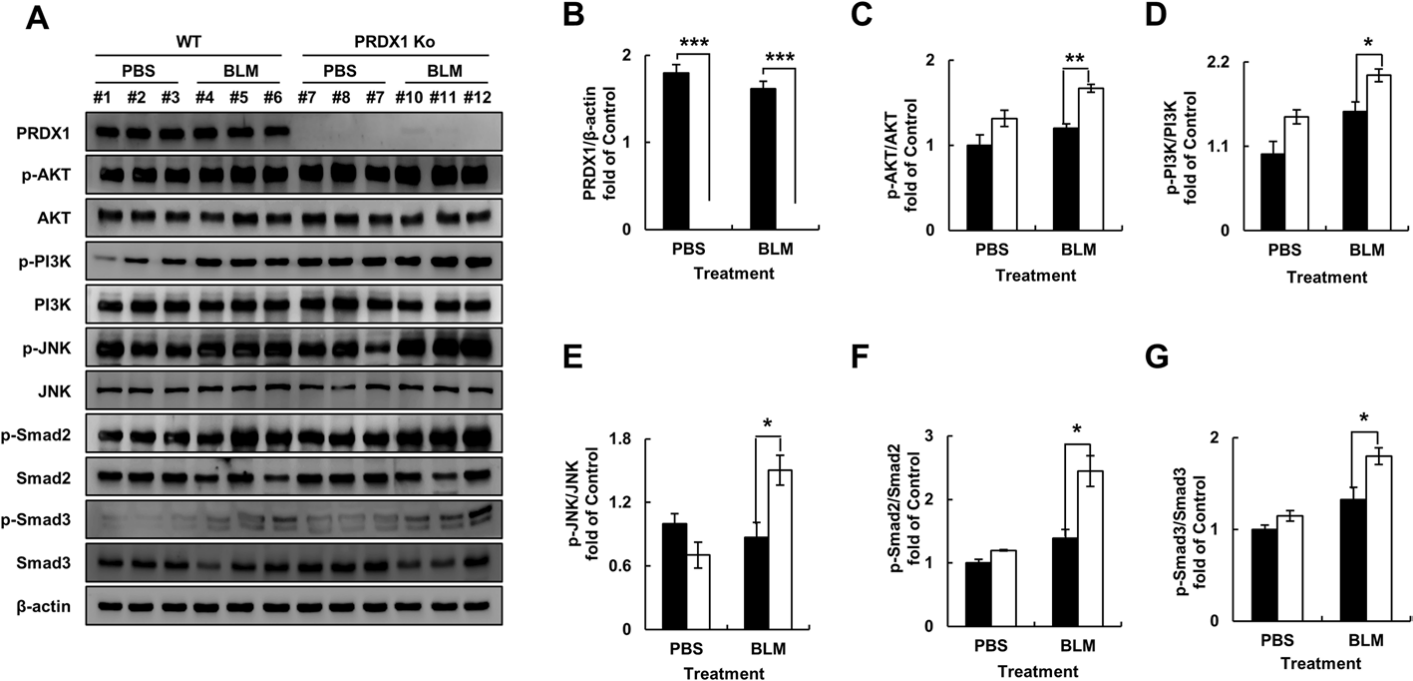
Expression of the PI3K/Akt and JNK/Smad signalling pathways-related proteins in mouse lung tissues. Lung fibrosis was induced in mice by intratracheal administration of BLM (5 mg/kgBW in 50 μL PBS) for 21 days. **A** Expression of the PI3K/Akt and JNK/Smad signalling pathways-related proteins in mouse lung tissues as determined by western blotting. **B**–**E** Quantitative analysis of the data presented in panel **A**. *, *p* < 0.05; **, *p* < 0.01; ***, *p* < 0.001. *PI3K* phosphoinositide 3-kinase, *Akt* protein kinase B, *JNK* c-Jun N-terminal kinase, *Smad* small of mothers against decapentaplegic, *PBS* phosphate-buffered saline

To further verify the role of the PI3K/Akt and JNK/Smad signalling pathways in the process of pulmonary fibrosis in mice, we assessed the phosphorylation of PI3K, Akt, JNK, and Smad in lung tissues by western blot analysis. The results showed that the phosphorylation levels of these proteins in the lung tissues of *PRDX1*-KO mice were significantly increased. The increases in the phosphorylation levels of Akt and PI3K were more pronounced; this may be due to the complex environment in the lung tissue, leading to differences between in vivo and in vitro phenotypes in mice (Fig. 8A–E). These results indicated that PRDX1 mainly regulates BLM-induced pulmonary fibrosis in mice through the PI3K/Akt and JNK/Smad signalling pathways.

## Discussion

Pulmonary fibrosis cannot be cured with medication, and the benefits of surgical lung transplantation are slim [42]; the hope for pulmonary fibrosis treatment is to find new therapeutic targets. BLM-induced pulmonary fibrosis model has become an important tool for studying the occurrence and development of pulmonary fibrosis diseases [43, 44]. BLM can induce an increase in ROS levels in A549 cells, increase oxidative stress, lead to mitochondrial damage, activate the EMT of epithelial cells, and trigger a pulmonary fibrosis cascade reaction [45]. PRDX1 can remove excessive cellular ROS and is involved in the occurrence and development of several lung diseases [4]. Research has shown that ROS are involved in the pathogenesis of most lung injuries. N-acetylcysteine is a ROS scavenger, and nebulized administration of N-acetylcysteine can alleviate BLM-induced pulmonary fibrosis in mice [46, 47]. In this study, the knockdown of PRDX1 induced a significant increase in ROS levels in lung epithelial cells and in the mitochondria, accompanied by mitochondrial damage (Fig. 1F), which laid the groundwork for ROS-induced damage to lung epithelial cells and triggered pulmonary fibrosis.

Myricetin has been found to improve BLM-induced pulmonary fibrosis by targeting HSP90β to inhibit TGF-β signalling [48]. Likewise, paeoniflorin has been shown to inhibit EMT-induced pulmonary fibrosis mediated by TGF-β1 through the Smad-dependent pathway [49]. BLM induces EMT in epithelial cells to promote the process of fibrosis via the TGF-β/Smad signalling pathway [50, 51]. PRDX1 has been demonstrated to regulate TGF-β-induced EMT through its antioxidant activity [52], pointing to the possibility that PRDX1 can participate in the TGF-β-induced EMT through its antioxidant activity in BLM-induced pulmonary fibrosis as well. In this study, in cells treated with BLM, PRDX1 knockdown induced significant changes in epithelial cell fibrosis morphology. PRDX1 knockdown significantly promoted the secretion of TGF-β1 by epithelial cells, increased the expression of the EMT-related proteins N-cadherin and Vimentin, decreased the expression of E-cadherin (Fig. 2B), and increased the expression of cell fibrosis related proteins SMA-α, Fibronectin, collagen I, and collagen III. These results indicate that PRDX1 knockdown can promote the EMT of epithelial cells and upregulate collagen expression. The increase in EMT can also explain the observed morphological changes and enhanced migration ability of lung epithelial cells. Significant changes in the levels of EMT-related proteins were detected in lung fibrosis tissues of BLM-treated PRDX1-KO mice, accompanied by significant changes in the expression of proteins involved in cell cycle and fibrosis, and a more severe process of pulmonary fibrosis was observed.

The damage of alveolar epithelial cells is an important starting point of pulmonary fibrosis, followed by the stimulation of fibroblast proliferation and collagen secretion, which is also an important part of the disease progression. Previous research has shown that ROS can induce fibroblast proliferation and collagen production by activating the PI3K/AKT pathway [53, 54]. BLM promotes the expression levels of cyclins A, D, E, PCNA, and other proteins in fibroblasts to promote cell proliferation. BLM also promotes the transition of cells from G0/G1 phase to G2/M phase and S phase to promote cell proliferation [55]. In this study, we found that after BLM treatment, PRDX1-KO cells, compared to WT cells, exhibited higher levels of increases in cellular and mitochondrial ROS, but no significant reduction was detected in mitochondrial membrane potential, which may be because the mitochondria of fibroblasts were not damaged. PRDX1 deficiency significantly increased the proliferation of fibroblasts by promoting the transition from G0/G1 phase to G2/M phase and induced the upregulation of cyclin B and the downregulation of cyclin P16 and P21, which is consistent with the above data. TGF-β can induce the differentiation of fibroblasts into myofibroblasts and enhance their migration ability; it is also the most studied extracellular matrix-stimulating factor in the process of fibrosis, including α-SMA and collagen I [56–58], which is consistent with the current observations. After BLM treatment, PRDX1 deficiency further promoted the secretion of TGF-β1 and the expression of α-SMA, Fibronectin, collagen I, and collagen III. PRDX1 knockdown also significantly enhanced the migration ability of fibroblasts.

The PI3K/AKT signalling pathway is one of the major signalling pathways that regulate various changes in cells. The activation of PI3K/AKT contributes to TGF-β-induced fibroblast aggregation, myofibroblast differentiation [28, 59], and α-SMA upregulation [29] in pulmonary fibrosis. The interaction of TGF-β and PI3K/AKT promotes the development of pulmonary fibrosis [60]. PRDX1 can interact with PTEN to activate downstream PI3K/AKT signalling and participate in other cellular signalling pathways [61, 62]. Moreover, BLM induces alveolar epithelial cell death through JNK-dependent activation of mitochondrial death pathway [63]. JNK1 is the main regulatory factor for TGF-β-induced EMT [64], and the stimulating effect of JNK in TGF-β-induced EMT requires the phosphorylation of Smad3 [65]. The key role of Smad2 and Smad3 is to regulate the transcription of TGF-β target genes [65]. In Smad3-KO mice, TGF-β cannot induce EMT and the key transcription factors of fibrosis, leading to a weakened pulmonary fibrosis phenotype [66]. PRDX1 reduces α-SMA expression by regulating JNK and inhibits fibroblast activation [67]. Our observations strongly suggest the important role of AKT/PI3K and JNK/Smad signalling pathways in the regulation of pulmonary fibrosis by PRDX1. In this study, PRDX1 knockdown promoted AKT/PI3K and JNK/Smad phosphorylation in epithelial cells. Pretreatments with LY and SP restored the phosphorylation levels of AKT/PI3K and JNK/Smad and inhibited EMT and the expression of fibrotic proteins. Furthermore, transcriptome analysis was performed to identify the signalling pathways involved in PRDX1-induced fibroblast proliferation and collagen secretion. The results indicated that PRDX1 deficiency significantly elevated the transcript levels of AKT/PI3K and JNK/Smad. PRDX1 knockout promoted AKT/PI3K and JNK/Smad phosphorylation in fibroblasts. After LY and SP pretreatments, AKT/PI3K and JNK/Smad phosphorylation levels were restored, and cell proliferation, cell cycle, and fibrotic proteins were inhibited. PRDX1 knockout resulted in an increase in AKT/PI3K and JNK/Smad phosphorylation levels in BLM-induced pulmonary fibrosis tissues in mice.

Mitochondria are crucial for pulmonary fibrosis in epithelial cells. In recent years, the endoplasmic reticulum (ER) has also become the focus of pulmonary fibrosis studies. Moreover, ER stress is thought to cause the injury and death of alveolar epithelial cells, which can aggravate the development of pulmonary fibrosis [68]. In addition, ER stress may play critical roles in the process of pulmonary fibrosis. Besides, ER stress and activation of the unfolded protein response have been reported to induce ROS production [69], and ROS are closely related to PRDX1 function. Therefore, whether PRDX1 can regulate the process of pulmonary fibrosis through ER stress needs further exploration. Studies have shown that miRNA species also play a significant role in regulating the occurrence and development of pulmonary fibrosis diseases [8]. However, the results of this did study not demonstrate how PRDX1 activates the PI3K/Akt and JNK/Smad signalling pathways, which requires further research. In future, bioinformatics websites, such as TargetScan, miRDB and miRTarBase, can be used together with the transcriptome sequencing results presented here to analyse the potential target mRNA functioning between PRDX1 and the PI3K/Akt and JNK/Smad signalling pathways, which will add value to this research’s findings.

## Conclusions

Overall, this study demonstrated that PRDX1 knockdown elevates ROS levels in epithelial cells, induces mitochondria damage, and promotes EMT, TGF-β1 secretion, and the activation of the JNK/Smad signalling pathway. Moreover, PRDX1 deficiency significantly promoted the proliferation of pulmonary fibroblasts and the secretion of collagen by increasing the activation of the PI3K/Akt and JNK/Smad signalling pathways, further exacerbating pulmonary fibrosis.

## Supplementary Information

**Additional file 1: Figure S1.** A. Quantitative analyses of the data presented in Fig.2 A. B. Quantitative analyses of the data presented in Fig.2 B. *, *p *< 0.05; **, *p *< 0.01; ***, *p *< 0.001. **Figure S2.** A. Quantitative analyses of the data presented in Fig.2 E. B. Quantitative analyses of the data presented in Fig.2 F. *, *p *< 0.05; **, *p *< 0.01; ***, *p *< 0.001. **Figure S3.** Quantitative analyses of the data presented in Fig.2G. *, *p *< 0.05; **, *p *< 0.01; ***, *p *< 0.001. **Figure S4.** A. Quantitative analyses of the data presented in Fig.4 C. B. Quantitative analyses of the data presented in Fig.4 D. C. Quantitative analyses of the data presented in Fig.4 H. *, *p *< 0.05; **, *p *< 0.01; ***, *p *< 0.001. **Figure S5.** A. Quantitative analyses of the data presented in Fig.5 A. B. Quantitative analyses of the data presented in Fig.5 B. C. Quantitative analyses of the data presented in Fig.5 C. *, *p *< 0.05; **, *p *< 0.01; ***, *p *< 0.001.

## Data Availability

The data that support the findings of this study are available on request from the corresponding author (Hu-Nan Sun), upon reasonable request.

## Abbreviations

PRDX: Peroxiredoxin
BLM: Bleomycin
ROS: Reactive oxygen species
EMT: Epithelial-mesenchymal transition
TGF-β: Transforming growth factor β
Smad: Small of mothers against decapentaplegic
α-SMA: α-Smooth muscle actin
JNK: C-Jun N-terminal kinase
WT: Wild type
Ko: Knockout
PI3K: Phosphoinositide 3-kinase
Akt: Protein kinase B
PBS: Phosphate-buffered saline
DMEM: Dulbecco’s modified Eagle’s medium
DHE: Dihydroethidium
Mito-SOX: Mitochondrial superoxide
P/S: Penicillin/streptomycin
TE: Trypsin/EDTA solution
FBS: Foetal bovine serum
MTT: 3-(4,5-Dimethylthiazol-2-yl)-2,5-diphenyltetrazolium bromide
DMSO: Dimethyl sulfoxide
ELISA: Enzyme-linked immunosorbent assay
FMLF: Foetal mouse lung fibroblasts
PI: Propidium iodide
H&E: Haematoxylin and eosin
ER: Endoplasmic reticulum
PCNA: Proliferating Cell Nuclear Antigen
