# Supplementary material for: PRDX1 negatively regulates bleomycin-induced pulmonary fibrosis via inhibiting the epithelial-mesenchymal transition and lung fibroblast proliferation in vitro and in vivo
Source: Cell Mol Biol Lett. 2023 Jun 2;28:48. doi: 10.1186/s11658-023-00460-x (PMC10236698; doi:10.1186/s11658-023-00460-x)
Supplement: Supplementary file 1 — Additional file 1: Figure S1. A. Quantitative analyses of the data presented in Fig.2 A. B. Quantitative analyses of the data presented in Fig.2 B. *, p < 0.05; **, p < 0.01; ***, p < 0.001. Figure S2. A. Quantitative analyses of the data presented in Fig.2 E. B. Quantitative analyses of the data presented in Fig.2 F. *, p < 0.05; **, p < 0.01; ***, p < 0.001. Figure S3. Quantitative analyses of the data presented in Fig.2G. *, p < 0.05; **, p < 0.01; ***, p < 0.001. Figure S4. A. Quantitative analyses of the data presented in Fig.4 C. B. Quantitative analyses of the data presented in Fig.4 D. C. Quantitative analyses of the data presented in Fig.4 H. *, p < 0.05; **, p < 0.01; ***, p < 0.001. Figure S5. A. Quantitative analyses of the data presented in Fig.5 A. B. Quantitative analyses of the data presented in Fig.5 B. C. Quantitative analyses of the data presented in Fig.5 C. *, p < 0.05; **, p < 0.01; ***, p < 0.001. [file 11658_2023_460_MOESM1_ESM.docx]

**Supplementary Figures**

**PRDX1 Negatively Regulates Bleomycin-Induced Pulmonary Fibrosis via Inhibiting the Epithelial-Mesenchymal Transition and Lung Fibroblast Proliferation in vitro and in vivo**


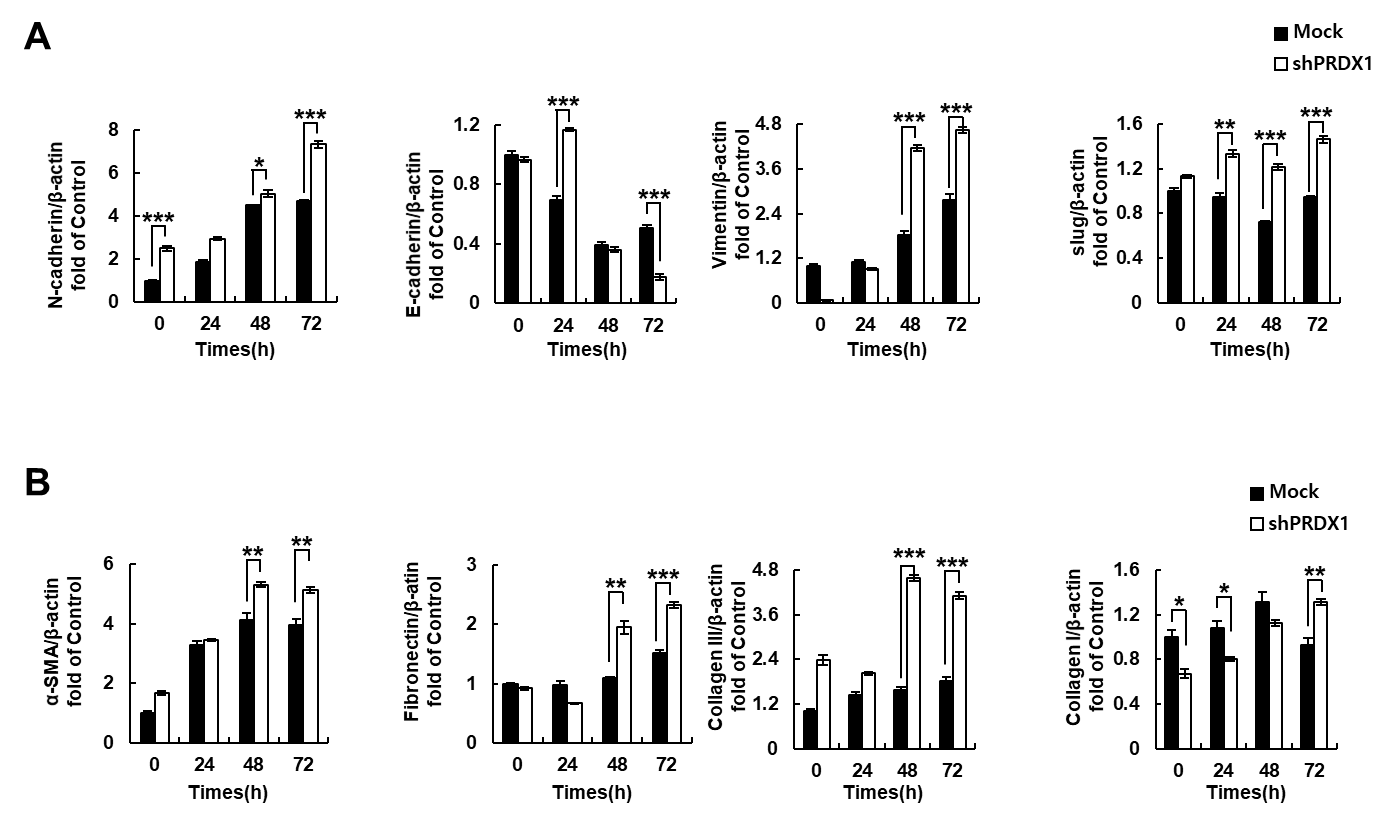


**Supplementary Figure S1.** **A.** Quantitative analyses of the data presented in Fig.2 A. **B.** Quantitative analyses of the data presented in Fig.2 B. ***, *p* < 0.05; ****, *p* < 0.01; *****, *p* < 0.001.

**
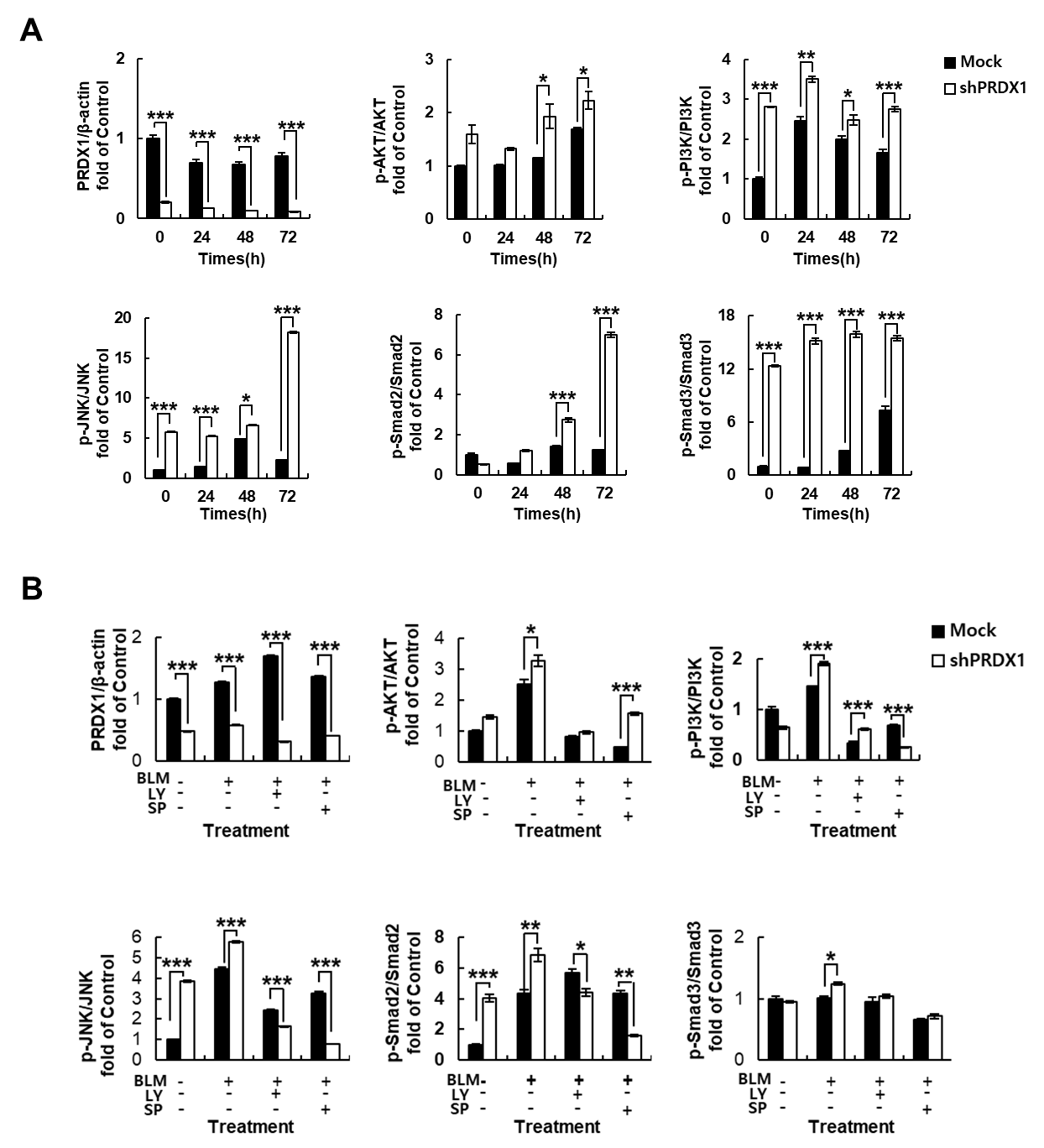
**

**Supplementary Figure S2.** **A.** Quantitative analyses of the data presented in Fig.2 E. **B.** Quantitative analyses of the data presented in Fig.2 F. ***, *p* < 0.05; ****, *p* < 0.01; *****, *p* < 0.001.

**
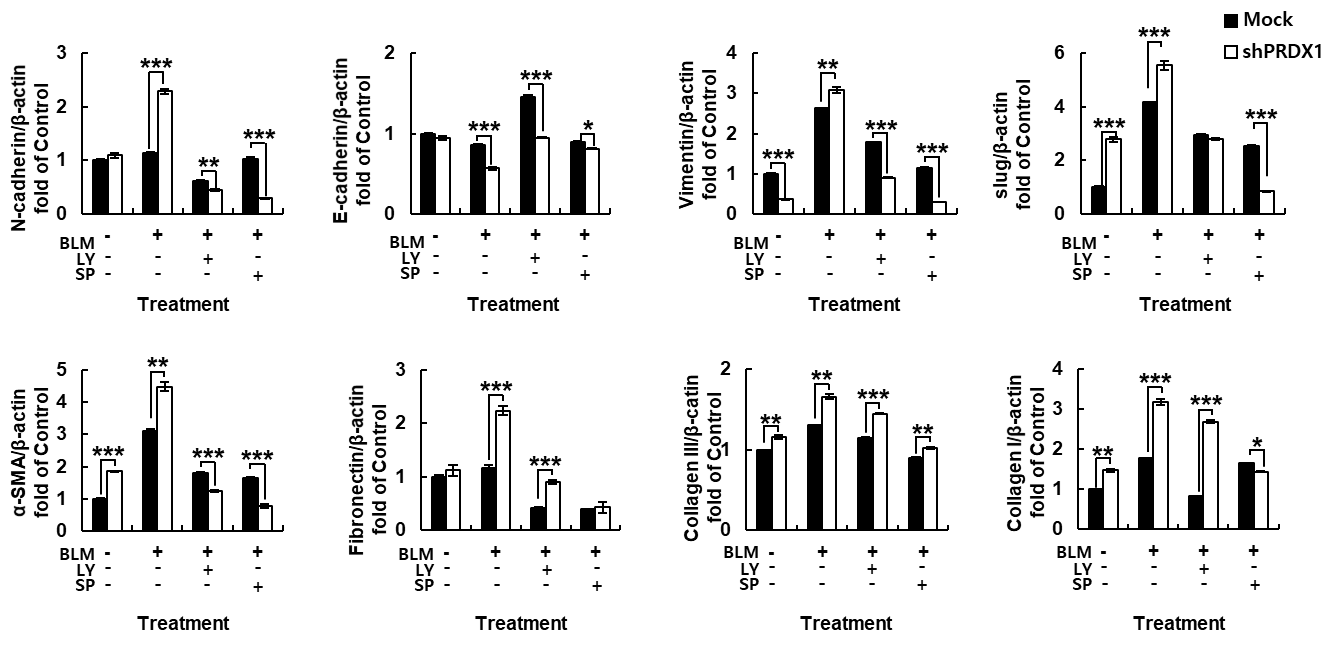
**

**Supplementary Figure S3.** Quantitative analyses of the data presented in Fig.2G. ***, *p* < 0.05; ****, *p* < 0.01; *****, *p* < 0.001.


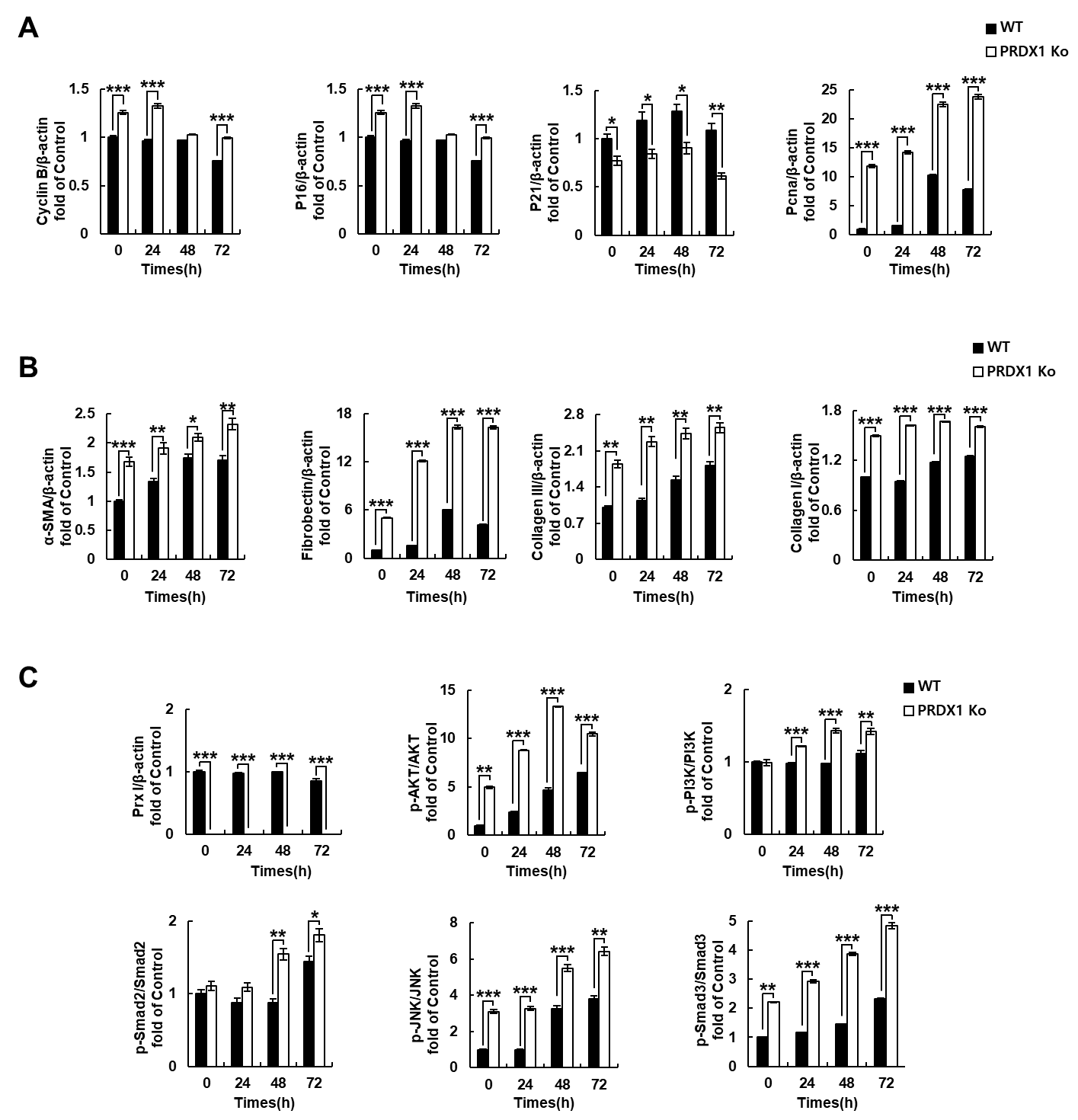


**Supplementary Figure S4.** **A.** Quantitative analyses of the data presented in Fig.4 C. **B.** Quantitative analyses of the data presented in Fig.4 D. **C.** Quantitative analyses of the data presented in Fig.4 H. ***, *p* < 0.05; ****, *p* < 0.01; *****, *p* < 0.001.


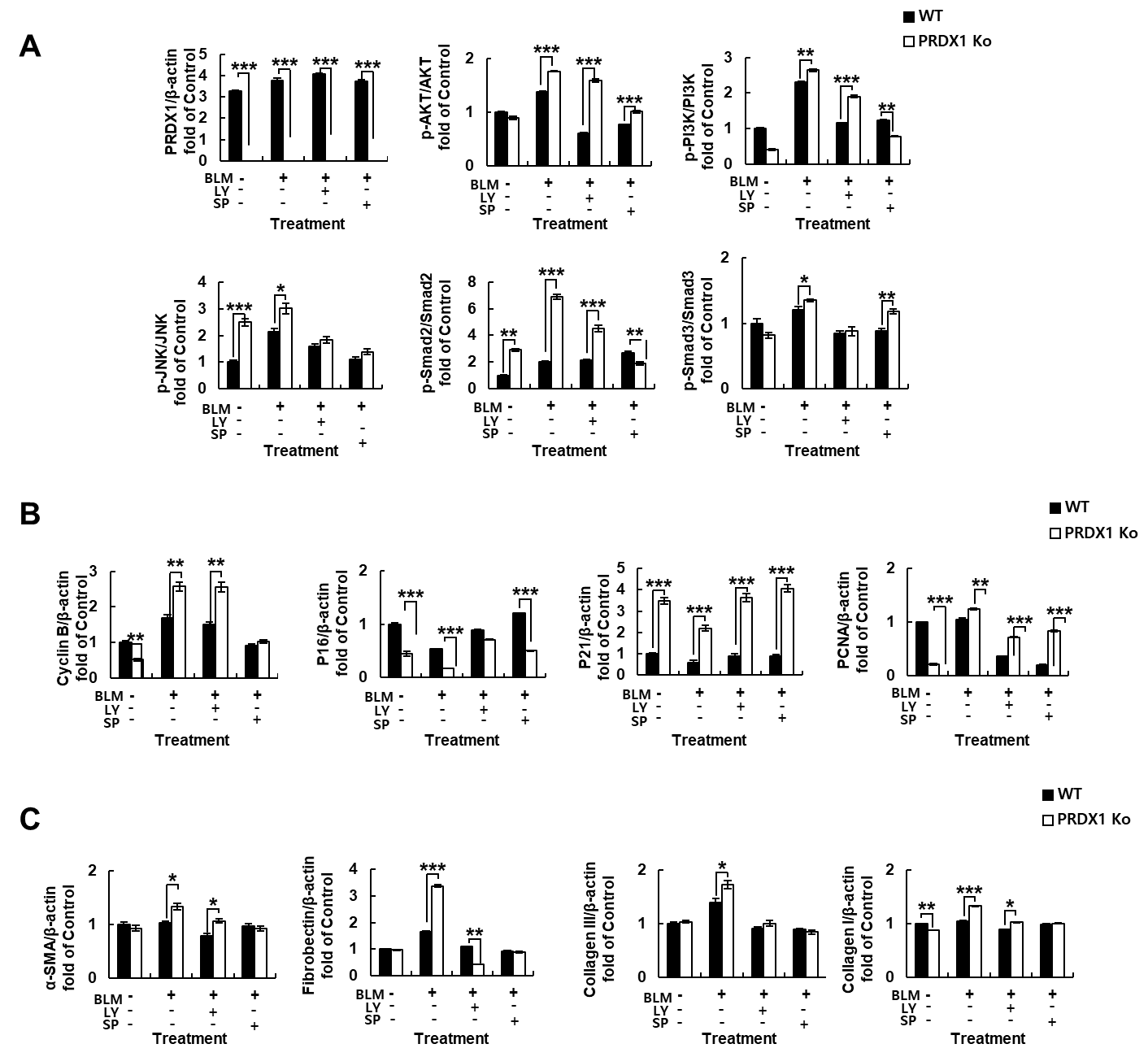


**Supplementary Figure S5.** **A.** Quantitative analyses of the data presented in Fig.5 A. **B.** Quantitative analyses of the data presented in Fig.5 B. **C.** Quantitative analyses of the data presented in Fig.5 C. ***, *p* < 0.05; ****, *p* < 0.01; *****, *p* < 0.001.
